# Prevalence of Germline Pathogenic Variants in Renal Cancer Predisposition Genes in a Population-Based Study of Renal Cell Carcinoma

**DOI:** 10.3390/cancers16172985

**Published:** 2024-08-27

**Authors:** Fiona Bruinsma, Philip Harraka, Susan Jordan, Daniel J. Park, Bernard Pope, Jason Steen, Roger L. Milne, Graham G. Giles, Ingrid Winship, Katherine M. Tucker, Melissa C. Southey, Tu Nguyen-Dumont

**Affiliations:** 1 Cancer Epidemiology Division, Cancer Council Victoria, East Melbourne, VIC 3002, Australia; fiona.bruinsma@cancervic.org.au (F.B.); roger.milne@cancervic.org.au (R.L.M.); graham.giles@cancervic.org.au (G.G.G.); 2 Burnet Institute, Melbourne, VIC 3004, Australia; 3 Centre for Epidemiology and Biostatistics, Melbourne School of Population and Global Health, The University of Melbourne, Melbourne, VIC 3010, Australia; 4 Precision Medicine, School of Clinical Sciences at Monash Health, Monash University, Clayton, VIC 3168, Australia; philip.harraka@monash.edu (P.H.); bjpope@unimelb.edu.au (B.P.); jason.steen@monash.edu (J.S.); tu.nguyen-dumont@monash.edu (T.N.-D.); 5 School of Public Health, The University of Queensland, Herston, QLD 4006, Australia; s.jordan@uq.edu.au; 6 Melbourne Bioinformatics, The University of Melbourne, Melbourne, VIC 3010, Australia; djp@unimelb.edu.au; 7 Department of Biochemistry and Pharmacology, University of Melbourne, Melbourne, VIC 3010, Australia; 8 Victoria Comprehensive Cancer Centre, The University of Melbourne Centre for Cancer Research, Melbourne, VIC 3010, Australia; 9 Department of Surgery, Royal Melbourne Hospital, Melbourne Medical School, The University of Melbourne, Melbourne, VIC 3010, Australia; 10 Department of Medicine, Melbourne Medical School, The University of Melbourne, Melbourne, VIC 3010, Australia; ingrid.winship@mh.org.au; 11 Genomic Medicine and Family Cancer Clinic, Royal Melbourne Hospital, Parkville, VIC 3052, Australia; 12 Hereditary Cancer Centre, Nelune Comprehensive Cancer Centre, Prince of Wales Hospital, Sydney, NSW 2031, Australia; kathy.tucker@health.nsw.gov.au; 13 Division of Medicine and Health, University of New South Wales, Sydney, NSW 2052, Australia; 14 Department of Clinical Pathology, Melbourne Medical School, The University of Melbourne, Melbourne, VIC 3010, Australia

**Keywords:** renal cell carcinoma, cancer predisposition, family history, population-based, gene panel testing

## Abstract

**Simple Summary:**

Studies of genetic predisposition to renal cell carcinoma (RCC) have been highly variable in design and have reported similarly highly variable rates of cases identified as carrying pathogenic variants in cancer susceptibility genes (4–26%). The aim of our study was to conduct a population-based study of genetic predisposition to RCC. We conducted a 21-gene panel sequencing study and identified that 18/1029 participants (1.7%) carried a pathogenic or likely pathogenic (PLP) variant. Genes with PLP variants included *BAP1*, *FH*, *FLCN*, *MITF*, *MSH6*, *SDHB*, *TSC1*, and *VHL*. This study provides further evidence that family history alone may not be sufficient for identifying all individuals who are at increased genetic risk of renal cell carcinoma, and further research is urgently needed to understand how to target genetic testing to identify those at high genetic risk of kidney cancer.

**Abstract:**

Renal cell carcinoma (RCC) has been associated with germline pathogenic or likely pathogenic (PLP) variants in recognised cancer susceptibility genes. Studies of RCC using gene panel sequencing have been highly variable in terms of study design, genes included, and reported prevalence of PLP variant carriers (4–26%). Studies that restricted their analysis to established RCC predisposition genes identified variants in 1–6% of cases. This work assessed the prevalence of clinically actionable PLP variants in renal cancer predisposition genes in an Australian population-based sample of RCC cases. Germline DNA from 1029 individuals diagnosed with RCC who were recruited through the Victoria and Queensland cancer registries were screened using a custom amplicon-based panel of 21 genes. Mean age at cancer diagnosis was 60 ± 10 years, and two-thirds (690, 67%) of the participants were men. Eighteen participants (1.7%) were found to carry a PLP variant. Genes with PLP variants included *BAP1*, *FH*, *FLCN*, *MITF*, *MSH6*, *SDHB*, *TSC1*, and *VHL*. Most carriers of PLP variants did not report a family history of the disease. Further exploration of the clinical utility of gene panel susceptibility testing for all RCCs is warranted.

## 1. Introduction

Renal cell carcinoma (RCC) accounts for 2% of cancer diagnoses worldwide and is associated with a mortality rate of 25% within 5 years [1]. Identification of those at greatest risk and early diagnosis through screening are important for improved survival because approximately 13% of cases are diagnosed with metastatic disease [2].

RCC aetiology is partially explained by hereditary factors, with 6% of cases reporting a family history of kidney cancer [3]. Several heritable renal cancer syndromes are clinically described (including von Hippel–Lindau Syndrome, Birt–Hogg–Dubé Syndrome, Hereditary Leiomyomatosis and RCC, Hereditary Papillary RCC, Cowden Syndrome, and Tuberous Sclerosis), all of which are associated with pathogenic or likely pathogenic (PLP) variants in genes that display an autosomal dominant inheritance.

Genetic epidemiology studies of RCC have been heterogenous in terms of design (hospital-based or commercial genetics service; retrospective or cross-sectional), the number of genes investigated (*n* = 19 to 808), and the prevalence of PLP variants observed (4–26%) [4,5,6,7,8,9,10,11]. These studies investigated cancer susceptibility genes that are not clinically actionable for RCC, including *CHEK2* and *BRCA2*. In studies that restricted their analysis to established RCC predisposition genes (*BAP1*, *EPCAM*, *FH*, *FLCN*, *MET*, *MITF*, *MLH1*, *MSH2*, *MSH6*, *PMS2*, *PTEN*, *SDHA*, *SDHB*, *SDHC*, *SDHD*, *TP53*, *TSC1*, *TSC2*, and *VHL*), PLP variants were identified in 1–6% of individuals tested [4,5,6,8,11,12]. However, these estimates may not reflect population estimates because the studies oversampled individuals with a suspected genetic aetiology for their RCC.

In Australia, there are 21 genes that are considered clinically actionable in the context of RCC by most clinical genetics services. These include genes associated with RCC syndromic diseases (*CDC73*, *FH*, *FLCN*, *MET*, *PTEN*, *SDHB*, *SDHC*, *SDHD*, *TSC1*, *TSC2*, and *VHL*), Lynch Syndrome (*EPCAM*, *MLH1*, *MSH2*, *MSH6*, *PMS2*), Li-Fraumeni Syndrome (*TP53*), and cancer predisposition genes (*BAP1*, *MITF*, *POLD1*, and *POLE*).

This study investigated the prevalence of PLP variants in these 21 genes in a population-based sample of RCC cases. The study provides estimates that are applicable to all individuals with RCC, irrespective of family history.

## 2. Materials and Methods

### 2.1. Study Participants

Participants were from the population-based family case-control component [13] of the Consortium for the Investigation of Renal Malignancies (CONFIRM) study. Participant characteristics are described in Table 1. Non-carriers were defined as individuals without a clinically actionable PLP variant in a gene included in our panel design.

Participants were recruited from the Victorian and Queensland Cancer Registries. The study included Australian residents diagnosed between 2009 and 2015 with a histologically confirmed RCC (ICD-10 C64) at age 18–74 years. Eligible participants had tumours at least 2cm in diameter, no previous diagnosis of RCC, and were able to complete the study questionnaires in English. 

Once an individual was identified as eligible by the cancer registry, the treating doctor was informed by the registry of the intention to invite the patient to participate in the study. A letter of invitation, participant information sheet, and study consent form were sent to the prospective participant, unless the treating doctor declined involvement of their patient.

### 2.2. Data and Biological Sample Collection 

Participants were asked to complete epidemiological questionnaires that included a section on family history of kidney cancer. Eighty-five percent of sequenced participants were recruited within 12 months of diagnosis. 

All participants were invited to provide a blood sample (or saliva if preferred) from which DNA was extracted for sequencing. 

### 2.3. Mutation Screening

Amplicon-based massively parallel sequencing of the coding regions and proximal intron–exon junctions of 21 genes (*BAP1*, NM_004656.4; *CDC73*, NM_024529.5; *EPCAM*, NM_002354.3; *FH*, NM_000143.4; *FLCN*, NM_144997.7; *MET*, NM_001127500.3; *MITF*, NM_000248.4; *MLH1*, NM_000249.4; *MSH2*, NM_000251.3; *MSH6*, NM_000179.3; *PMS2*, NM_000535.7; *POLD1*, NM_002691.4; *POLE*, NM_006231.4; *PTEN*, NM_000314.8; *SDHB*, NM_003000.3; *SDHC*, NM_003001.5; *SDHD*, NM_003002.4; *TP53*, NM_000546.6; *TSC1*, NM_000368.5; *TSC2*, NM_000548.5, and *VHL*, NM_000551.4) was performed on germline-derived DNA from 1071 individuals using the Hi-Plex protocol (Nguyen-Dumont et al., 2015 Breast Cancer Res Treat). Massively parallel sequencing (150 bp paired-end) was performed on a MiSeq (Illumina, San Diego, CA, USA). Mapping to the human reference build hg19 and variant calling were performed as described in Nguyen-Dumont et al., 2015 [14]. A DNA sample was considered to have been successfully sequenced when at least 80% of the targeted regions were covered at a read depth of at least 50X.

### 2.4. Variant Prioritisation and Classification

Variant annotation and filtering were performed using the variant analysis software Varseq (version 2.3.0, Golden Helix, Bozeman, MT, USA), consistent with those applied in exome/genome studies. Variants were selected for further investigation if they were considered genuine (observed at a total read depth ≥ 50X and a variant allele fraction of ≥0.2) and rare (minor allele frequency < 0.01, or absent, in gnomAD exomes and genomes). Variants were considered PLP if they were classified in ClinVar (accessed 2 March 2023) as PLP with a review status of at least “2 stars”. When variants were classified as PLP in ClinVar with a review status of “1 star”, or were absent from ClinVar but predicted to be loss-of-function, then variant classification was performed according to the American College of Medical Genetics (ACMG) criteria [15], including updated recommendations by the Association for Clinical Genomic Science [16], the Cancer Variant Interpretation Group UK [17], and the ClinGen Sequence Variant Interpretation Working Group [18,19,20]. 

PLP variants were manually assessed using Integrative Genomics Viewer (IGV) for errors in alignment, and sequencing or mapping artefacts were excluded. All PLP variants were validated by Sanger sequencing at the Australian Genome Research Facility or the Garvan Institute for Medical Research (Sydney, Australia).

The number of rare variants of uncertain significance (VUSs) in this sample of participants was calculated by counting the number of variants that were neither classified as (i) PLP according to the criteria described above, nor (ii) benign or likely benign according to ClinVar (at least “1 star”). These variants were not inspected in IGV.

### 2.5. Statistical Analyses

All *p*-values were calculated using Fisher’s exact test; a *p*-value <0.05 was considered significant.

## 3. Results

Of the 3113 individuals diagnosed with RCC who were invited to participate in CONFIRM, 1424 provided consent. Of those, 1071 had germline DNA available for screening, and 1029 were successfully sequenced with an average of 97% of the targeted regions covered at a minimum of 50X. Mean age (±standard deviation) at cancer diagnosis was 60 ± 10 years, and two-thirds (690, 67%) were men (Table 1). 

Eighteen cases (1.7%) were found to carry a PLP variant (Table 2). The mean age at diagnosis of RCC for these cases was 58 ± 10 years and eleven (61%) were men (Table 1). The gene with the most PLP variant carriers identified was *MITF* with five individuals carrying NM_000248.4:c.952G>A; p.Glu318Lys (Table 2). Other genes with PLP variants included *FLCN*, *FH*, *MSH6*, *VHL*, *BAP1*, *SDHB*, and *TSC1*. No PLP variants were identified in *CDC73*, *EPCAM*, *MET*, *MLH1*, *MSH2*, *PMS2*, *POLD1*, *POLE*, *PTEN*, *SDHC*, *SDHD*, *TP53*, or *TSC2*. Three hundred and forty-six VUSs were identified in 289 participants (28%).

Family history data were available for 897 (87%) of the 1029 participants (15 PLP carriers, 882 non-carriers), of whom 769 reported if any of their first-degree relatives had kidney cancer (12 PLP carriers, 757 non-carriers), and 752 reported if any of their second-degree relatives had kidney cancer (12 PLP carriers, 740 non-carriers). Of PLP variant carriers, only 1 of 12 (8%) had a first-degree relative with kidney cancer compared with 54 of 757 (7%) non-carriers (*p* = 0.59). Also, 1 of 12 (8%) PLP carriers had a second-degree relative with kidney cancer compared with 34 of 740 (5%) non-carriers (*p* = 0.44) (Table 1). These two PLP carriers who reported a family history (carriers 4 and 7, Table 2) both had a PLP variant in *FLCN*. In total, 86 of 882 (10%) non-carriers had a family history of RCC.

Histopathological presentations for PLP variant carriers included seven (39%) with clear cell RCC, four (22%) with chromophobe RCC, three (17%) with papillary RCC, and four (22%) with RCC (not otherwise specified) (Table 2). Presentations for non-PLP variant carriers included 606 (60%) with clear cell RCC, 96 (9%) with chromophobe RCC, 107 (11%) with papillary RCC, 186 (18%) with RCC (not otherwise specified), and 15 (1%) with other subtypes of RCC.

## 4. Discussion

This study provides an important population-based assessment of the prevalence of clinically actionable PLP germline variants in RCC in Australia. PLP variants were identified in 1.7% of participants, consistent with previous studies that identified 1–6% were carriers of PLP variants in an RCC predisposition gene [4,5,6,8,11,12]. Unlike most previous studies, this study did not oversample for individuals with a likely genetic aetiology [7,8,11,12], or recruit from clinical settings that limit analysis and relevance to a subset of the population [4,6,9,10].

PLP variants were identified in 8 of 21 established RCC predisposition genes included in our panel design, with variants most commonly observed in *MITF*. The PLP variant *MITF* p.Glu318Lys was observed in 0.5% of participants and has been previously reported to be associated with an increased risk of RCC [21]. In a retrospective study, Nguyen et al., 2017 reported this variant in 0.7% of 1235 individuals with kidney cancer, the majority of whom did not report a family history of kidney cancer [12]. Although there are no well-described clinical features of RCC associated with *MITF* p.Glu318Lys, the histopathology predominantly observed with this variant is either clear cell or papillary RCC [12].

Most individuals who were identified as carrying a PLP variant had no reported family history of RCC, indicating that they, and possibly their relatives, are unaware of their genetic predisposition to RCC. In Australia, genetic counselling and germline panel testing are recommended for individuals with a RCC diagnosis who have a suspected genetic aetiology based on their clinical presentation, including age of onset and family history of RCC. Genetic testing offered more broadly may therefore be useful to identify those at genetically increased risk of RCC, irrespective of family history. Furthermore, predictive testing for PLP variants may be offered to family members to enable them to engage in clinical care adapted to their hereditary risk, of which the family were hitherto unaware.

Conversely, most participants (98%) who reported a family history were not identified as carrying a PLP variant, so the possible hereditary cause of their RCC remains undetermined. It is possible these individuals carry a variant in a gene that is not yet demonstrated to be associated with RCC. Indeed, a recent report from Yngvadottir et al., 2022 provided some evidence for an association between RCC risk and PLP variants in *CHEK2*, which was not included in our panel design [22].

The prevalence of PLP variants in the 21 genes included in this study could be higher than reported here. The estimate of VUS in this study was high (*n* = 346) due to the difficulty in interpreting these variants; most were rare missense variants with currently insufficient evidence of pathogenicity for classification as PLP.

For instance, classification of a splice variant, BAP1 NM_004656.4:c.783+2T>C, was challenging under current ACMG guidelines. This variant is located in the donor splice site of intron 9. The in silico tool, SpliceAI, predicted a cryptic donor site 2bp downstream, which, if engaged, creates a transcript with four additional nucleotides resulting in a frameshift event. This prediction was used in the assessment of the variant for PVS1 and to support the LP classification of the variant. However, donor site GT>GC variants can be problematic because an estimated 15–18% are partially functional and give rise to normal transcripts [23]. It is also unclear to what extent adjacent splice site motifs could influence splicing in this region. In the future, RNA sequencing may be useful to assess the potential spliceogenic impact of this variant.

Two variants classified as VUSs in this study, according to current ACMG criteria, were classified as PLP in ClinVar with a review status of 1 star. These were in *FH* (NM_000143.4:c.1196G>A; p.Ser399Asn) and *SDHD* (NM_003002.4:c.278_280dupATT; p.Tyr93dup). Neither ClinVar entry provided sufficient details about affected participants to be able to curate these variants as PLP according to current ACMG standards.

The significant strength of this study is its population-based design; however, because individuals were recruited from cancer registries rather than clinical settings, we do not have detailed clinical information about features that could possibly accompany the genetic syndromes and assist variant classification, especially for variants identified in *FLCN*, *FH*, and *TSC1*.

Some types of genomic variation cannot be detected by the gene panel design and technology applied, including deep intronic, UTR, structural, or copy-number variants. This limitation is true for many technologies applied in previous studies, and thus our understanding of these types of genomic variants in these genes is likely to be incomplete. However, large deletions (10–13 kb) have been identified in *VHL* in cohorts of RCC cases [22] that, if present, remain undetected in our study. It is also likely that further RCC susceptibility genes exist that have not yet been identified/validated and thus were not included in this study (e.g., *CHEK2*) [22].

Furthermore, not all individuals with RCC reported in the registries were eligible, nor consented, to participate in this study. Some individuals may have declined participation because they had worse health outcomes that were associated with carrying a PLP variant.

## 5. Conclusions

This study indicates that the proportion of individuals with RCC who would benefit from short-read gene panel testing is less than 2%. This study provides further evidence that (i) family history alone may not be sufficient for identifying all individuals who are at increased genetic risk of RCC and (ii) further research is urgently needed to identify and characterise additional genetic risk factors for RCC.

## Figures and Tables

**Table 1:** Characteristics of cases with renal cell carcinoma participating in CONFIRM.

	PLP Variant Carriers	Non-Carriers	All Tested Individuals (100%)
Participants: N (%)	18 (1.7%)	1011 (98.3%)	1029
Sex: N (%)			
Male	11 (2%)	679 (98%)	690
Female	7 (2%)	332 (98%)	339
Age at diagnosis: mean ± SD	58 ± 10	60 ± 10	60 ± 10
Histology: N (%)			
Clear cell	7 (1%)	606 (99%)	613
Chromophobe	4 (4%)	96 (96%)	100
Papillary	3 (3%)	107 (97%)	110
Not otherwise specified	4 (2%)	186 (98%)	190
Other	0	15 (100%)	15
Unknown	0	1 (100%)	1
Family history: N (%)			*p*-value
First-degree relative with RCC			*p* = 0.59
Yes	1 (2%)	54 (98%)	
No	11 (2%)	703 (98%)	
Missing	6 (2%)	254 (98%)	
Second-degree relative with RCC			*p* = 0.44
Yes	1 (3%)	34 (97%)	
No	11 (2%)	706 (98%)	
Missing	6 (2%)	271 (98%)	

**Table 2:** Characteristics of carriers of pathogenic or likely pathogenic variants in CONFIRM.

Carrier	Gene	HGVS c. ^b^	HGVS p. ^b^	Sex	Age of Diagnosis (Years)	Affected First-Degree Relative	Affected Second-Degree Relative	Histology
1	*BAP1*	NM_004656.4:c.783+2T>C		Female	54	Unknown	Unknown	clear cell
2	*FH*	NM_000143.4:c.698G>A	p.Arg233His	Male	54	No	No	NOS
3	*FH*	NM_000143.4:c.575C>T	p.Pro192Leu	Male	56	Unknown	Unknown	clear cell
4 ^a^	*FLCN*	NM_144997.7:c.1432+1G>A		Male	66	Yes	Unknown	papillary
5 ^a^	*FLCN*	NM_144997.7:c.1432+1G>A		Male	47	Unknown	Unknown	chromophobe
6	*FLCN*	NM_144997.7:c.1318_1334dup	p.Leu449Glnfs*25	Female	62	No	No	NOS
7	*FLCN*	NM_144997.7:c.469_471delTTC	p.Phe157del	Female	62	No	Yes	chromophobe
8	*MITF*	NM_000248.4:c.952G>A	p.Glu318Lys	Male	63	Unknown	No	papillary
9	*MITF*	NM_000248.4:c.952G>A	p.Glu318Lys	Male	74	No	No	clear cell
10	*MITF*	NM_000248.4:c.952G>A	p.Glu318Lys	Male	60	Unknown	No	clear cell
11	*MITF*	NM_000248.4:c.952G>A	p.Glu318Lys	Male	72	No	No	clear cell
12	*MITF*	NM_000248.4:c.952G>A	p.Glu318Lys	Female	73	No	Unknown	NOS
13	*MSH6*	NM_000179.3:c.3469G>A	p.Gly1157Ser	Male	56	Unknown	No	chromophobe
14	*MSH6*	NM_000179.3:c.2057G>A	p.Gly686Asp	Female	45	No	No	papillary
15	*SDHB*	NM_003000.3:c.505C>T	p.Gln169Ter	Male	47	No	Unknown	NOS
16	*TSC1*	NM_000368.5:c.589delT	p.Cys197Alafs*13	Female	58	No	No	chromophobe
17	*VHL*	NM_000551.4:c.548C>A	p.Ser183Ter	Female	67	No	No	clear cell
18	*VHL*	NM_000551.4:c.556G>T	p.Glu186Ter	Male	35	No	No	clear cell

^a^ Variant carriers 4 and 5 are first-degree relatives (parent–child). ^b^ Variant nomenclature according to the Human Genome Variation Society (HGVS), HGVS.c for coding DNA, and HGVS.p for protein variants.

## Data Availability

The data presented in this report can be requested via PEDIGREE. https://www.cancervic.org.au/research/epidemiology/pedigree.
